# Retrospective evaluation of an integrated molecular-epidemiological approach to cyclosporiasis outbreak investigations – United States, 2021

**DOI:** 10.1017/S0950268823001176

**Published:** 2023-07-19

**Authors:** Lauren Ahart, David Jacobson, Marion Rice, Travis Richins, Anna Peterson, Yueli Zheng, Joel Barratt, Vitaliano Cama, Yvonne Qvarnstrom, Susan Montgomery, Anne Straily

**Affiliations:** 1 Division of Parasitic Diseases and Malaria, Center for Global Health, Centers for Disease Control and Prevention, Atlanta, GA, USA; 2 Oak Ridge Institute for Science and Education, Oak Ridge, TN, USA; 3 Eagle Global Scientific, San Antonio, TX, USA

**Keywords:** Molecular epidemiology, Food-borne infections, Cyclospora

## Abstract

Cyclosporiasis results from an infection of the small intestine by *Cyclospora* parasites after ingestion of contaminated food or water, often leading to gastrointestinal distress. Recent developments in temporally linking genetically related *Cyclospora* isolates demonstrated effectiveness in supporting epidemiological investigations. We used ‘temporal-genetic clusters’ (TGCs) to investigate reported cyclosporiasis cases in the United States during the 2021 peak-period (1 May – 31 August 2021). Our approach split 655 genotyped isolates into 55 genetic clusters and 31 TGCs. We linked two large multi-state epidemiological clusters (Epidemiologic Cluster 1 [n = 136 cases, 54 genotyped] and Epidemiologic Cluster 2 [n = 42 cases, 15 genotyped]) to consumption of lettuce varieties; however, product traceback did not identify a specific product for either cluster due to the lack of detailed product information. To evaluate the utility of TGCs, we performed a retrospective case study comparing investigation outcomes of outbreaks first detected using epidemiological methods with those of the same outbreaks had TGCs been used to first detect them. Our study results indicate that adjustments to routine epidemiological approaches could link additional cases to epidemiological clusters of cyclosporiasis. Overall, we show that CDC’s integrated genotyping and epidemiological investigations provide valuable insights into cyclosporiasis outbreaks in the United States.

## Background

More than 1400 laboratory-confirmed cases of cyclosporiasis were reported to the U.S. Centers for Disease Control and Prevention (CDC) in each of 2018, 2019, and 2020, reflecting an increase in cyclosporiasis incidence since 2015 [1–3]. Cyclosporiasis infections occur after ingesting produce and/or water contaminated by *Cyclospora* [4], resulting in a range of nonspecific symptoms including watery diarrhoea, loss of appetite, cramps, and bloating [5]. Epidemiological investigations of cyclosporiasis outbreaks can be challenging due to an incubation period between consumption of contaminated produce and symptom onset that may last up to 2 weeks and intermittent remission/relapse of symptoms that may delay seeking care, and because *Cyclospora* often contaminates commonly consumed fresh produce that is served in mixtures with other produce (e.g., leafy greens, berries, and herbs) or as garnishes [5]. These circumstances make it difficult for case patients to recall specific produce items consumed potentially weeks prior to interview by a public health official, which hinders traceback investigations due to lack of highly detailed exposure information (i.e., product brand names). Sensitive molecular tools that identify clusters of genetically related *Cyclospora* infections could abate these challenges by allowing epidemiological follow-up investigations to focus on case patients infected with *Cyclospora*, or a closely related *Cyclospora* strain, which should increase the likelihood of uncovering the suspect food vehicle [6].

The CDC uses the Cybernetic Clustering Of Nonclonal Eukaryotes (CYCLONE) bioinformatic workflow [2] to identify infections caused by genetically related *Cyclospora* isolates. CYCLONE identifies genetic clusters with a ‘haplotype-based’ approach due to high heterozygosity and genetic complexities found in *Cyclospora* isolates [7]. Previous studies have evaluated CYCLONE’s performance by comparing the composition of genetic clusters to analogous epidemiological clusters, the latter being used as a set of expected clustering outcomes [1, 3]. These evaluations demonstrate that genetic clusters produced by CYCLONE have over 96% agreement with epidemiological clusters. CYCLONE has been historically used to support the existence of epidemiologically identified outbreaks by demonstrating genetic relationships among isolates from case patients linked within an epidemiological cluster [3]. Starting in 2020, the CDC began to use temporal information to prioritise genetically defined clusters for epidemiological follow-up. Temporal-genetic clusters (TGCs) are informative because produce items contaminated with *Cyclospora* are likely to be sources of infection for a limited range of time, rather than sporadically over many months, due to the limited shelf-life of fresh produce.

Genetic clustering data are used to inform epidemiological investigations in a variety of applications [8], and PulseNet has used this approach to identify foodborne and waterborne outbreaks in the United Stated for over 20 years [9]. In practice, the PulseNet model of outbreak investigation triggers epidemiological investigations after the identification of illness clusters caused by genetically similar pathogen isolates. Short laboratory turnaround times are critical for detecting clusters as long delays may make it difficult for epidemiologists to identify common exposures. Recent developments have shortened the processing time of whole genome sequencing (WGS), leading to improved genetic resolution generated in a similar time to pulse field gel electrophoresis [10–12]. Currently, cyclosporiasis investigations do not follow the PulseNet model because epidemiological investigations do not solely rely on the identification of a genetic cluster. We believe there is value in assessing how cyclosporiasis outbreak investigations would fit into a PulseNet approach, which may improve the integration of CYCLONE’s genetic clustering outputs with epidemiological investigations.

The objective of this study was to retrospectively investigate how the PulseNet outbreak investigation model might have performed had it been applied during the 2021 U.S. cyclosporiasis peak-period. This analysis represents an important step in determining whether an approach that uses genetic data to lead epidemiological follow-up (i.e., the PulseNet model) would benefit cyclosporiasis outbreak investigations. Ultimately, these results will help determine how operationalisation of *Cyclospora* genotyping data can improve future cyclosporiasis outbreak investigations and facilitate the translation of genotyping data into actionable public health recommendations.

## Methods

### Two-step analysis approach

We first detail the genetic clustering results and describe the relationship between TGCs and epidemiological clusters in 2021, with a focus on the two largest TGCs (2021_003 and 2021_005) and two multi-state epidemiological clusters (Epidemiologic Cluster 1 and Epidemiologic Cluster 2). Second, we focus our retrospective epidemiological investigations on a subset of isolates possessing identical genotypes to explore how a genetics-first approach, similar to PulseNet, would perform in cyclosporiasis investigations.

### Faecal isolates

U.S. state health departments (SHDs) submitted stool specimens from laboratory-confirmed *Cyclospora*-positive case patients to the CDC for genotyping. Upon receipt at the CDC, the specimens were de-identified and given a unique identifier. DNA was extracted from the stool specimens following previously described methods [13], and isolates with a Ct value greater than 38 after quantitative polymerase chain reaction (qPCR) targeting the *Cyclospora* 18S rRNA gene [13] were excluded from downstream laboratory processing as isolates with Ct > 38 are highly unlikely to yield genotypes that pass CYCLONE’s inclusion criteria (Supplementary Material). In the remaining isolates, eight *Cyclospora* genotyping markers were PCR-amplified and sequenced following previously described methods [3]. New York and Texas SHDs, along with the Public Health Agency of Canada (PHAC), generated sequencing data in-house following the protocols described earlier and submitted their data to the CDC to be analysed in CYCLONE. CYCLONE genotyping is detailed in the Supplementary Material.

### Cluster identification using the CYCLONE workflow

Genotyped isolates must meet the following inclusion criteria to be included in clustering analysis: haplotypes identified in any combination of 5 of the 8 markers, or haplotypes from 4 markers if at least 3 of those 4 were CYCLONE’s high-entropy markers [2, 14]. Distance matrix calculation for the isolates passing the inclusion criteria (n = 655) was performed alongside a genetically diverse reference population of 1169 genotypes generated between 2018 and 2020 [1–3]. Cluster membership of isolates was determined using a recently described statistical framework for genetically linking pairs of isolates and applying a stringency setting of 99.5% [15]. Descriptions of the algorithms underpinning distance matrix calculation and genetic clustering are provided in the Supplementary Material. Isolates in the same genetic cluster were temporally linked by examining collection dates of each specimen to identify TGCs [1]. Initial TGCs are created when three or more isolates within the same genetic cluster originate from two or more jurisdictions, and the isolates have collection dates within fourteen days of each other; isolates are added to an existing TGC if they have collection dates that fall within seven days of the most recently collected isolate in the TGC. The hierarchically clustered tree was visualised using the GGTREE R package [16, 17].

### Epidemiological data collection and cluster investigations

Epidemiological data received by the CDC primarily consist of responses to the Cyclosporiasis National Hypothesis Generating Questionnaire (CNHGQ), or state-adapted versions of the CNHGQ, which are used by epidemiologists at SHDs to interview case patients. The CNHGQ captures the clinical history, travel history, produce exposures, and information on where produce items were purchased or consumed. The CDC focused investigations on reported cases with no history of international travel during the 14 days prior to the illness onset during the U.S. cyclosporiasis peak-period, meaning that their illness was likely acquired within the United States (i.e., domestically acquired cases).

Epidemiological data reported via the CNHGQ were matched to genotyped isolates by case ID and analysed to detect emerging epidemiological clusters. We noted commonalities in produce exposures, grocery stores, or restaurants reported by 50% or more of case patients in a TGC. This analysis allowed us to focus investigation efforts on the most likely contaminated produce commodity, recognising that many fresh produce items included in the CNHGQ follow a seasonal pattern and are commonly consumed during the summer months. Data completeness was also assessed, and requests were made to the appropriate SHD if pertinent epidemiological data were missing (i.e., CNHGQ and/or Case ID). TGCs with over 50% of case patients reported as lost to follow-up (i.e., SHD was not able to complete an interview) were considered to have an insufficient amount of data for epidemiological review. Finally, we excluded case patients who reported history of international travel during the 14 days prior to the illness onset. Throughout the article, we use capital letters to signify epidemiologic clusters (e.g., Epidemiologic Cluster 1) and lowercase letters to signify epidemiologic exposures (e.g., lettuce type 1).

### Examination of epidemiological links among isolates with identical genotypes: PulseNet model Case Study

We selected isolates from a subset of genetic clusters to assess how genotyping data could be used to guide epidemiological investigations. First, we filtered out any genetic clusters with less than two epidemiologically linked isolates. Next, we identified and retained genetically identical isolates within the remaining genetic clusters by comparing each isolate’s genotype to the consensus genotype for the genetic cluster. The consensus genotype consists of every haplotype found in over 50% of all isolates within a genetic cluster. We then analysed epidemiological data for completeness and excluded isolates with missing associated epidemiological data. Finally, we compared produce exposures for genetically identical isolates within each cluster to existing epidemiological clusters.

## Results

### Cyclosporiasis peak-period summary

**Figure 1.**
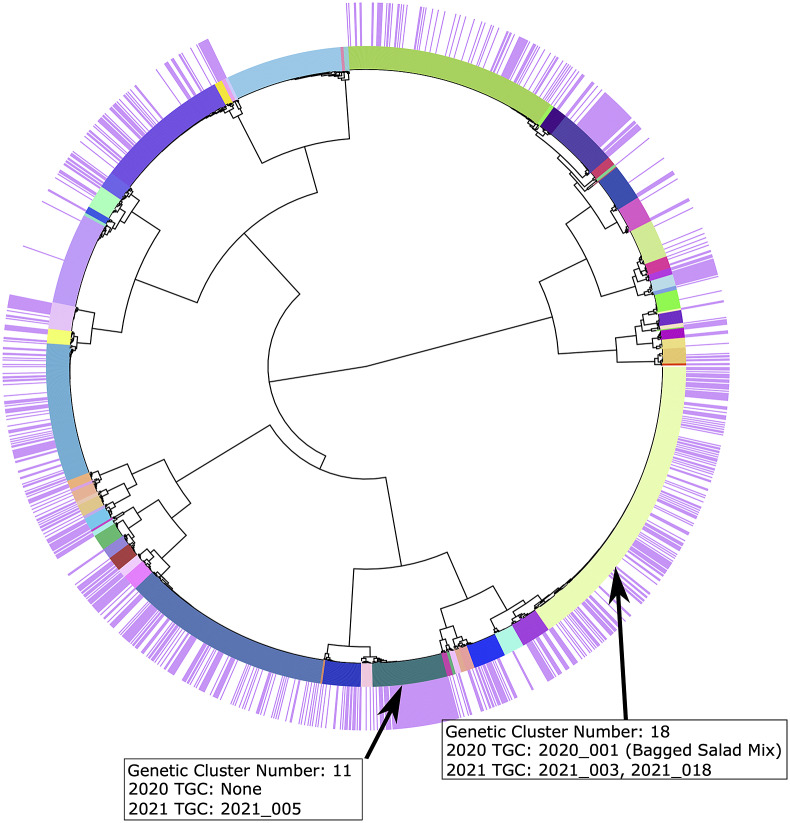
Tree representing genetic cluster memberships. Each colour on the inner ring represents a distinct genetic cluster. The specimens highlighted in purple in the outer ring are from 2021, and the specimens highlighted in white in the outer ring are reference specimens from 2018 to 2020. The locations of TGC 2021_003 (Genetic Cluster 18) and TGC 2021_005 (Genetic Cluster 11) are annotated by text boxes. Genetic cluster 18 contains TGC 2020_001 from 2020, which was linked to the bagged salad mix outbreak.

Between 1 May and 31 August 2021, the CDC was notified of 1123 laboratory-confirmed cyclosporiasis cases from 36 U.S. states and New York City. Data from 797 isolates were submitted to the CDC for genotyping: 502 isolates were sequenced at the CDC and 295 were isolates sequenced by domestic and international partner laboratories. Genotypes were obtained from 655 of the 797 isolates, which were distributed across 55 genetic clusters (Figure 1). Isolates that did not yield a genotype (n = 142) either did not pass the qPCR inclusion criteria (n = 68) or did not have haplotypes identified in a sufficient combination of markers to pass the inclusion criteria (n = 74). A total of 44 TGCs were detected during the peak-period; however, 13 TGCs dissolved due to shifts in genetic cluster membership as new genotypes were added to the dataset each week (this phenomenon is discussed elsewhere [1]). This resulted in 561 isolates belonging to 31 distinct TGCs spread across 23 genetic clusters at the end of 2021 (two or more TGCs were found in seven genetic clusters).

Fourteen of the 31 (45%) TGCs identified contained insufficient case data (Table 1) for epidemiological review, and all but one (2021_007) of the TGCs with insufficient data possessed six or fewer isolates. Fifty-nine isolates belonged to TGC 2021_007, yet over half of case patients within this TGC (n = 36, 61%) reported a history of international travel, were lost to follow-up, or missing pertinent information (Table 1). The remaining TGCs with sufficient epidemiological data available to review (n = 17, 55%) varied in size, and 10 of 17 (59%) TGCs were associated with epidemiological clusters (Table 1). Findings from a secondary retrospective analysis of the most commonly reported food exposures across all 17 TGCs showed that case patients who reported cucumber, tomatoes, or berries also reported consuming a salad, suggesting that herbs, cucumbers, tomatoes, and berries may have been consumed with a leafy green product. Of the 17 TGCs with sufficient epidemiological data to review, 14 TGCs included case patients who reported a history of international travel, were lost to follow-up, or missing epidemiological data (Table 1).

**Table 1.** All TGCs detected in the 2021 cyclosporiasis peak-period

TGC code (TGCs that comprise the same genetic cluster/strain)	No. of genotypes in TGC	Epidemiological cluster name (No. of genotyped isolates)	International travel destination(s): No. of case patients	No. of lost to follow-up case patients (%)	Missing pertinent epidemiological data (i.e., case ID or CNHGQ^a^), %
2021_001 (2021_002)	3	n/a^b^	Unknown: 1	0 (0%)	2 (67%)
2021_002 (2021_001)	54^c^	n/a^b^	Mexico: 11 Unknown: 4	3 (6%)	10 (19%)
2021_003 (2021_018)	129^c^	Epidemiologic Cluster 1 (n = 5) Epidemiologic Cluster 2 (n = 13) Local Cluster 1 (n = 2)	Mexico: 1 Unknown: 8	13 (10%)	12 (9%)
2021_004 (2021_026)	4	n/a^b^	Unknown: 3	0 (0%)	1 (25%)
2021_005	70	Epidemiologic Cluster 1 (n = 27)	Unknown: 2	2 (3%)	2 (3%)
2021_006	56	Epidemiologic Cluster 1 (n = 10) Local Cluster 2 (n = 2)	n/a^b^	4 (7%)	5 (9%)
2021_007 (2021_012)	59^c^	Local Cluster 3 (n = 1)	Mexico: 13 Unknown: 8	3 (5%)	12 (20%)
2021_008 (2021_031)	6^c^	n/a^b^	Unknown: 1	1 (17%)	1 (17%)
2021_012 (2021_007)	4^c^	n/a^b^	n/a^b^	0 (0%)	2 (50%)
2021_014	43	Local Cluster 4 (n = 2) Lettuce 1 (n = 1)	Unknown: 1	2 (5%)	2 (5%)
2021_015 (2021_037)	19	Local Cluster 5 (n = 2) Epidemiologic Cluster 1 (n = 6)	Unknown: 1	0 (0%)	1 (5%)
2021_017	3	n/a^b^	Mexico: 1 Unknown: 1	0 (0%)	1 (33%)
2021_018 (2021_003)	3	n/a^b^	n/a^b^	0 (0%)	2 (67%)
2021_019 (2021_020, 2021_029)	6	n/a^b^	Mexico: 3	0 (0%)	1 (17%)
2021_020 (2021_019, 2021_029)	3	n/a^b^	n/a^b^	0 (0%)	0 (0%)
2021_022	3	n/a^b^	Unknown: 2	1 (33%)	0 (0%)
2021_023	16	n/a^b^	Mexico: 1 Unknown: 1	2 (13%)	3 (19%)
2021_026 (2021_004)	4	n/a^b^	Unknown: 1	0 (0%)	1 (25%)
2021_029 (2021_019, 2021_020)	10	n/a^b^	Mexico: 1 Unknown: 2	0 (0%)	1 (10%)
2021_031 (2021_008)	4	Epidemiologic Cluster 1 (n = 1)	n/a^b^	0 (0%)	1 (25%)
2021_034	16	Epidemiologic Cluster1 (n = 1) Epidemiologic Cluster 2 (n = 2)	Mexico: 1 Unknown: 1	0 (0%)	2 (13%)
2021_035	5	n/a^b^	n/a^b^	0 (0%)	2 (40%)
2021_036	4	n/a^b^	Mexico: 1	0 (0%)	0 (0%)
2021_037 (2021_015)	7	Local Cluster 7 (n = 6)	n/a^b^	0 (0%)	0 (0%)
2021_038	3	Epidemiologic Cluster 1 (n = 1)	n/a^b^	0 (0%)	0 (0%)
2021_039	4	n/a^b^	n/a^b^	1 (25%)	0 (0%)
2021_040	3^c^	n/a^b^	Unknown: 1	0 (0%)	1 (33%)
2021_041	3	n/a^b^	Mexico: 1	0 (0%)	2 (67%)
2021_042	10	Epidemiologic Cluster 1 (n = 2)	n/a^b^	0 (0%)	2 (20%)
2021_043	3	n/a^b^	n/a^b^	1 (33%)	1 (33%)
2021_044	4	n/a^b^	n/a^b^	2 (50%)	2 (50%)

Abbreviations: TGC, temporal-genetic cluster; CNHGQ, Cyclosporiasis National Hypothesis Generating Questionnaire.

a Cyclosporiasis national hypothesis generating questionnaire.

b Not available.

c Isolates submitted by the public health agency of Canada.

### Cyclosporiasis epidemiologic cluster investigations

In 2021, two major multi-state epidemiological clusters were investigated: Epidemiologic Cluster 1 and Epidemiologic Cluster 2. The Epidemiologic Cluster 1 investigation began in July following a notice from epidemiologists at SHDs that several case patients reported a specific lettuce type (lettuce type 1) from a single product brand. CDC epidemiologists used preliminary findings provided by state partners to analyse data reported via the CNHGQ; two different types of lettuce emerged as suspect vehicles after a thorough review of all leafy green exposures reported by case patients. A single product brand (or affiliated brands) was more frequently named by case patients who reported lettuce type 1; thus, we defined Epidemiologic Cluster 1 cluster to include only case patients reporting lettuce exposure from this product brand or an affiliate brand. Epidemiologic Cluster 1 consisted of 136 case patients from 20 states. Most case patients resided in the northeastern and midwestern regions of the United States. A total of 54 (40%) case patients in Epidemiologic Cluster 1 had a successfully genotyped isolate, and those isolates were associated with multiple genotypes and nine different TGCs (Table 2); however, half of the isolates belonged to TGC 2021_005 (n = 27, 50%).

**Table 2.** Multi-state epidemiological clusters identified in 2021 and their associations with identified TGCs

Epidemiological cluster name	Number of laboratory-confirmed cases	Total number of successfully genotyped isolates	Associated TGC codes	Number of successfully genotyped isolates	Number of successfully genotyped isolates with dual exposures^a^	Number of isolates with an identical genotype^b^
Epidemiologic Cluster 1	136	54	2021_003	5	1	2
			2021_005	27	3	21
			2021_006	10	1	n/a^c^
			2021_014	1	0	n/a^**c**^
			2021_015	6	0	n/a^**c**^
			2021_031	1	0	n/a^**c**^
			2021_034	1	0	n/a^**c**^
			2021_038	1	0	n/a^**c**^
			2021_042	2	0	n/a^**c**^
Epidemiologic Cluster 2	42	15	2021_003	13	1	8
			2021_034	2	0	n/a^**c**^

Abbreviations: TGC, temporal-genetic cluster.

a A dual exposure was defined as a reported exposure to both lettuce type 1 and lettuce type 2.

b Identical to the consensus genotype of their respective TGC.

c Not available.

Epidemiologic Cluster 2 was identified in August after CDC epidemiologists noted the emergence of a second lettuce type (lettuce type 2) that had not been previously reported by U.S. cyclosporiasis case patients. Lettuce type 2 did not appear to be associated with a particular product brand, and most case patients epidemiologically linked to Epidemiologic Cluster 2 resided in the southern United States. In total, 42 case patients were epidemiologically linked across 10 states. A total of 15 (36%) case patients in Epidemiologic Cluster 2 had a successfully genotyped isolate (Table 2). This cluster was associated with two TGCs (2021_003 and 2021_034), but 87% of epidemiologically linked cases (n = 13) were assigned to TGC 2021_003 (Table 2). The genetic cluster containing TGC 2021_003 possessed reference genotypes from isolates previously linked to a bagged salad mix outbreak from 2020 [1] (Figure 1). However, case patients epidemiologically linked to the 2020 bagged salad mix outbreak did not report the same lettuce exposure as case patients in the 2021 Lettuce 2 cluster.

Five case patients with successfully genotyped isolates reported exposures to both lettuce type 1 and lettuce type 2 (i.e., ‘dual exposures’). CDC epidemiologists linked these case patients to either Epidemiologic Cluster 1 or Epidemiologic Cluster 2 based on similarity to genotypes within a TGC and case patients’ geographical clustering with other case patients who already had been epidemiologically linked to Epidemiologic Cluster 1 or Epidemiologic Cluster 2 (Table 2). Notably, the four case patients with dual exposures within TGCs 2021_005 and 2021_006 reported purchasing lettuce type 1 from four distinct grocery stores.

In addition to the two multi-state outbreaks, epidemiologists at SHDs in eight states identified nine other single-state epidemiological clusters, yielding a total of 228 laboratory-confirmed cyclosporiasis cases linked to an epidemiological cluster. Of these, 84 cases had isolates that were successfully genotyped. The CDC received at least one specimen for genotyping from case patients associated with six of the nine clusters, and all genotyped isolates belonging to the same single-state epidemiological cluster also belonged to the same TGC.

### Epidemiological investigation of isolates with an identical genotype: the PulseNet model case study

The retrospective PulseNet model analysis focused on isolates with identical genotypes within the two TGCs that had the majority of isolates linked to Epidemiologic Cluster 1 and Epidemiologic Cluster 2 to determine whether additional isolates could be linked to these epidemiological clusters. After excluding isolates as described in the Methods, 185 isolates met the inclusion criteria for our PulseNet model case study. This analysis allowed us to investigate why these case patients were not linked to an epidemiological cluster despite their genetic similarity to isolates from other case patients epidemiologically linked to a cluster.

Within TGC 2021_003, 53 of 129 (41%) genotypes were identical to the consensus genotype. Of the 53 isolates with an identical genotype, 11 (21%) were from case patients linked to Epidemiologic Cluster 1 (n = 2), Epidemiologic Cluster 2 (n = 8), and Local Cluster 1 (n = 1) (Table 3). The remaining identical genotypes in TGC 2021_003 (n = 42, 78%) could not be epidemiologically linked to a cluster due to one of the following reasons: missing epidemiological data (n = 6, 14%), reported history of international travel (n = 3, 7%), lack of product information (n = 14, 33%), or failure to meet the epidemiological cluster inclusion criteria (n = 19, 45%) (Table 3). In summary, the primary reasons for not linking the remaining case patients to Epidemiologic Cluster 1 or Epidemiologic Cluster 2 in TGC 2021_003, despite the genetically identical genotypes, were the lack of detailed product information available (i.e., brand name) or case patients failed to meet the epidemiological cluster inclusion criteria. The single isolate in TGC 2021_003 with dual epidemiological exposures did not have a complete genotype, so it was excluded from the analysis of identical genotypes.

**Table 3.** Primary TGCs and epidemiological cluster classifications

TGC code	Total number of isolates	Number of genetically identical isolates	Total number of case patients yielding identical genotypes	Epidemiological cluster name (number of case patients)	Justification for why case patients were not epidemiologically linked to a cluster (number of case patients)
2021_003	129	53	11	Epidemiologic Cluster 1 (n = 2)	n/a^**a**^
				Epidemiologic Cluster 2 (n = 8)	
				Local Cluster 1 (n = 1)	
			42	n/a^**a**^	*Missing exposure and/or epidemiological data* Lost to follow-up (n = 5) Could not recall produce exposures (n = 1)
					*Travel history* History of international travel within 14 days prior to illness onset (n = 3)
					*Lack of product information* Salad product from restaurant containing lettuce type 1, unknown product brand (n = 1) Purchased lettuce type 1, unknown product brand (n = 13)
					*Failed to meet epidemiological cluster inclusion* Salad product did not contain lettuce type 1or lettuce type 2 (n = 2) Other bagged salad or leafy green exposure (n = 17)
2021_005	70	46	21	Epidemiologic Cluster 1	n/a^**a**^
			25	n/a^**a**^	*Missing exposure and/or epidemiological data* Lost to follow-up (n = 2)
					*Lack of product information* Purchased lettuce type 1, unknown product brand (n = 8)
					*Failed to meet epidemiological cluster inclusion* Exposure to lettuce type 1, product brand not of interest (n = 1) Did not report any leafy green exposures (n = 3) Did not report a specific leafy green exposure (n = 11)

Abbreviations: TGC, temporal-genetic cluster.

a Not available.

A total of 46 isolates with identical genotypes were identified in TGC 2021_005. Of these, 21 (46%) case patients were associated with Epidemiologic Cluster 1, while the remaining 25 (54%) case patients could not be epidemiologically linked to a cluster (Table 3). Two case patients (8%) were missing epidemiological information, 8 (32%) lacked product information, and 15 (60%) failed to meet the epidemiological inclusion criteria (Table 3). Three case patients in TGC 2021_005 reported exposures to both lettuce type 1 and lettuce type 2 but were linked to epidemiological clusters based on their isolate’s genotype: two had an identical genotype to other isolates in 2021_005 and were linked to Epidemiologic Cluster 1, while the remaining isolate did not have data from all eight genotyping markers and was excluded from the identical genotype analysis.

## Discussion

Rapid and effective public health response to cyclosporiasis outbreaks can be improved with a better understanding of how cases are linked, both genetically and epidemiologically. The primary objective of this study was to evaluate an approach that has the potential to enhance identification and investigation of cyclosporiasis clusters by prioritising *Cyclospora* isolates with identical genotypes. This two-step procedure first identified isolates in TGCs that belonged to the same epidemiological cluster and then retrospectively analysed genetically identical isolates in a subset of genetic clusters to see if additional epidemiologically linked isolates could be identified.

Nearly half of all cases linked to TGCs either reported international travel during the 2-week incubation period when exposures would have likely occurred or lacked pertinent epidemiological data (i.e., missing CNHGQ and/or Case ID), making it difficult to link case patients to epidemiological clusters. In some instances, epidemiological data were available, but investigations remained challenging due to the high number of case patients reporting more than one fresh produce exposure. Nevertheless, we focused on leafy green exposures as most other fresh produce items were rarely reported without a leafy green co-exposure.

Isolates linked to the two leafy green-associated multi-state epidemiological clusters primarily belonged to the two largest TGCs identified in 2021 (Epidemiologic Cluster 1: 2021_005 and Epidemiologic Cluster 2: 2021_003); however, nine different TGCs were identified among case patient isolates associated with Epidemiologic Cluster 1, suggesting that either a single lettuce product might have been contaminated with multiple strains of *Cyclospora*, or that multiple lettuce products might have been contaminated with different strains of *Cyclospora.* Product traceback investigations for commonly consumed food items remain difficult to conclude, especially without specific epidemiological data. Thus, the exact contamination scenario of Epidemiologic Cluster 1 could not be determined. Nearly all epidemiologically linked isolates in Epidemiologic Cluster 2 were assigned to the same TGC (2021_003), yet, similar to Epidemiologic Cluster 1, product traceback investigations were inconclusive. Several case patients epidemiologically linked to Epidemiologic Cluster 2 cluster reported shopping at a single grocery store chain that did not have a shopper card system. Investigators find shopper card records (i.e., receipts) useful to gather information on the types or brands of produce purchased by case patients. These records are particularly useful for cyclosporiasis investigations where the time between consumption of a product and interview by local or state health departments is weeks or even months after potential exposure, which can affect a person’s ability to accurately recall what they ate or retrieve any purchase information for foods consumed. Without product information, product traceback investigations are hindered, and it is difficult to identify a single producer, as was the case for Epidemiologic Cluster 2.

The identical genotype retrospective study provided insights regarding why certain case patients were not linked to an epidemiological cluster. These data are important for understanding how using the genotyping data as a primary trigger for conducting epidemiological investigations (i.e., the PulseNet model) would impact cyclosporiasis investigations. We found most case patients could not be epidemiologically linked to a cluster because of incomplete epidemiological data, recent international travel, not specifying a product brand for produce exposures, or exposure to a produce item not associated with a known epidemiological outbreak. This pilot analysis indicates that starting analyses with TGCs would have likely resulted in similar epidemiological cluster membership compared to the approach currently used by CDC epidemiologists as case patients cannot be linked to an epidemiological cluster without sufficient epidemiological data. However, using a PulseNet approach to investigate these clusters might have resulted in more complete epidemiological data because secondary case patient interviews would have been conducted after identification of genetic linkages, and supplemental questionnaires could have been used to target specific brands or produce exposures.

An important caveat to using genetic data to trigger epidemiological investigations is the time between initial diagnosis of *Cyclospora* infection, receipt of faecal specimens at the CDC, and determination of genotyping results. CDC laboratory turnaround time is less than 10 days, but some of this process is highly variable and could impact real-time cluster detection as a result. In addition, the lag between the consumption of a contaminated produce item, onset of symptoms, sample collection, and submission to the CDC could be several weeks; thus, it is important that SHDs still perform epidemiologic investigations while genetic data are analysed at the CDC in order to maximise case patients’ dietary recall. Subsequently, exploratory interviews should be conducted on a subset of case patients within each TGC (i.e., after genotyping) to gather specific information on product brands. This two-pronged approach maintains the current practice of gathering epidemiologic data soon after diagnosis, while also implementing a PulseNet-like approach, where a subset of case patients are interviewed after genetic clustering is complete. We believe such a model would enhance traceback investigations via the collection of more detailed information from case patients.

Epidemiological data remain necessary for identifying the source of cyclosporiasis outbreaks as relying solely on *Cyclospora* genetic data can limit investigations. For example, not all laboratory-confirmed cases of cyclosporiasis have a specimen sent to the CDC for genotyping and some isolates do not yield a useable genotype, both of which contribute to an underrepresentation of the true number of outbreak cases. Moreover, epidemiological data are typically available to review before genetic data are processed, and epidemiologists at SHDs often identify an epidemiological cluster before isolates or sequence data are submitted to the CDC (e.g., during interviews with case patients). Perhaps most importantly, genetic clustering results alone are of limited value; to become actionable (e.g., to initiate a product recall), a common food vehicle must be identified through an epidemiological investigation. Efforts to improve epidemiological data collection and enhance epidemiological investigations are ongoing. Most recently, CDC epidemiologists revised the CNHGQ (version 3.4) to include supplementary questions that capture additional details about leafy green products [1, 2]. Additionally, a new, stand-alone question regarding the suspect vehicle identified in Epidemiologic Cluster 2 was added to the CNHGQ; historically, this exposure was rarely reported by cyclosporiasis cases but warranted further consideration following the Epidemiologic Cluster 2 investigation. These revisions should allow for improved identification of specific produce items during outbreak investigations, which will in turn also help inform product traceback investigations led by the U.S. Food and Drug Administration (FDA).

In this study, we observed that most isolates genotyped from case patients associated with the two multi-state epidemiological clusters identified in 2021 belonged to one of the two major TGCs. About half of all genotyped isolates epidemiologically linked to Epidemiologic Cluster 1 were assigned to TGC 2021_005, while the remainder were distributed across multiple TGCs. The reason for this outcome is not clear; however, it might be due to case patients misremembering food exposures (i.e., reporting lettuce type 1 by mistake) or to the possibility that multiple sources of *Cyclospora* contaminated this type of produce during the U.S. cyclosporiasis peak-period. We cannot rule out the possibility that lettuce type 1 was merely identified as a suspect vehicle because it is commonly consumed during the cyclosporiasis peak-period, which highlights our call for enhanced epidemiological data collection. The analysis of isolates with an identical genotype allowed us to conclude that non-epidemiologically linked case patients with isolates genetically identical to genotypes of other epi-linked case patients were not misclassified, but rather could not be classified at all because they were missing epidemiological data, lacked sufficient details on produce exposures, or did not report the produce exposure of interest. Before the CYCLONE genetic clustering data can be operationalised like the PulseNet model, CDC epidemiologists would potentially need to adjust how cyclosporiasis clusters are defined and how case patients are interviewed. In summary, this study highlights the continued benefits of an integrated genetic and epidemiological investigation approach, while providing avenues to enhance public health investigations and response to cyclosporiasis outbreaks.

## Supporting information

Ahart et al. supplementary material Ahart et al. supplementary material

## Data Availability

FASTQ sequence data for all isolates analysed in this study are available under NCBI BioProject PRJNA578931. Readers may contact the authors for access to the CYCLONE code used in this study’s bioinformatic analysis.

## Supplementary material

For supplementary material accompanying this paper visit http://doi.org/10.1017/S0950268823001176. click here to view supplementary material
