# Supplementary material for: Retrospective evaluation of an integrated molecular-epidemiological approach to cyclosporiasis outbreak investigations – United States, 2021
Source: Epidemiol Infect. 2023 Jul 19;151:e131. doi: 10.1017/S0950268823001176 (PMC10540164; doi:10.1017/S0950268823001176)
Supplement: Ahart et al. supplementary material [file S0950268823001176sup001.docx]

**Data to support Ct value cutoff.**

Specimens genotyped at CDC from 2019 and 2020 with a Ct value > 38 most often yielded 0 or 1 genotyping markers (Figure 1). A minimum of 4 or 5 markers (depending on the markers present) is required to pass genotyping in CYCLONE and therefore specimens with a Ct value of 38 (red dashed line on plot) are unlikely to yield successful genotypes if they were sequenced. In interest of saving time and money resources, specimens with a Ct value > 38 were not sequenced at CDC or partner labs in the 2021 peak period. Our data suggest a more aggressive Ct cutoff of 35 cycles could be used; however, we chose a Ct cutoff of 38 to conservatively allow for low Cyclospora-load samples the chance to yield data from a sufficient number of markers.

**Supplemental Figure 1**

**CYCLONE Genotyping extended methods**

FASTQs are entered into CYCLONE, where adapter sequences are trimmed and reads are merged with the BBTools analysis suite [1]. The trimmed and merged reads are mapped, with BWA [2], against two databases to determine which workflow the reads will enter. The first database contains haplotypes from the Mt. Junction marker (‘Junction-haplotypes’ workflow) and the second database contains haplotypes from the seven other markers (‘Non-junction haplotypes’ workflow).

*‘Non-junction haplotypes’ Workflow*

Mapped reads are aligned against all known *Cyclospora* haplotypes from a manually curated database using bowtie2 and samtools [3, 4]. Each of the 7 non-junction markers are bioinformatically split into approximately 100bp fragments (aka marker parts) to reduce the impact of PCR-induced chimeras on haplotype identification. The nucleotide position of each marker part is annotated in a BED file and we use tools from jvarkit (samjs, pcrclipreads, biostar84452) [5] to extract fragments from the sample’s reads that align to each marker part. We note the depth of reads mapping to each marker part to calculate cutoffs used later in the workflow

The reads mapping to each marker part from each sample are clustered individually at 100% identity using CD-HIT [6]. We use 100% identity because there may be multiple haplotypes at a single marker part in a single specimen (i.e., heterozygosity). We blast each cluster against our known haplotypes to determine if there are any novel haplotypes discovered, and if the potential novel haplotypes pass our novel haplotype required depth*. If the novel haplotypes have sufficient depth, they are written to our haplotype database.

After the detection of novel haplotypes from all samples, we BLAST [7] our clustered reads against all known haplotypes to determine which haplotypes are found in each sample at each marker part. The blast parameters are -perc_identity 100 -qcov_hsp_perc 100 -num_threads -max_target_seqs 1 -word_size 7 -dust no -soft_masking false. We compare the depth of reads in the blast output file to the required depth for identification of haplotypes in a sample’s genotype**, if there is sufficient depth of coverage, we concatenate the clustered read into a single FASTA file per sample. We then perform a final blast step of all known haplotypes against each sample’s FASTA file from the previous step to confirm which haplotypes are present in each sample. The final two blast steps are performed to ensure we are detecting full length haplotypes with sufficient depth in each sample.

The resulting haplotypes from the 7 non-junction markers are combined with the haplotypes detected in the Junction-haplotypes workflow. The combined haplotypes file (i.e., a specimen’s genotype) for all specimens are then combine into a single haplotype datasheet. The haplotype datasheet contains genotypes for all specimens being analyzed in the current outbreak period, as well as the genotypes for all reference specimens. The haplotype datasheet is used as input for distance matrix calculation and clustering.

*In the ‘Junction-haplotypes’ workflow*

Identification of Junction haplotypes is an iterative process. We first take the mapped reads and search for the forward and reverse primers to ensure that the full junction marker is being extracted. The extracted reads are clustered with CD-HIT and MIRA is used to assemble the clustered sequences and adapters are cut from the assembled contig using cutadapt. Bowtie2, samtools, and bcftools are then used to align sample reads against the assembled contig and create a consensus sequence. This process is repeated, with multiple steps of cutting adapters to make sure the ending sequence reflects a true biological sequence. The depth of each final consensus sequence is calculated after blasting against a database of known junction haplotypes to determine if the haplotype passes the depth required for novel haplotypes* (if a novel haplotype has been detected) or the depth required for identification of a haplotype that is already part of the reference database**.

*Novel haplotype required depth.

Haplotypes must > 100x depth of coverage or make-up > 25% of the sample’s reads at the marker part where the haplotype was discovered.

**Sample genotype required depth.

Haplotypes must > 20x depth of coverage or make-up > 10% of the sample’s reads at the marker part where the haplotype was discovered.

The exact parameters used for each bioinformatic tool in each step are not listed due to length of the code; however, CYCLONE code is available via CDC’s GitHub in limited release. Please contact the corresponding author (DJ) to request CYCLONE software.

**CYCLONE Clustering extended methods**

*Distance Matrix*

The distance matrix calculation steps are described in the supplemental text in Nascimento et al. 2020 [8]. In brief, the presence/absence of each haplotype is compared between each pair of specimens in the dataset. The probability that any two specimens are related is calculated by examining the number of shared haplotypes, which is weighted by the frequency of the shared haplotypes in the full dataset and the Shannon entropy for each locus where haplotypes are shared (i.e., if two specimens share very rare haplotypes, this is a good indication they are closely related, while sharing common haplotypes contributes little to the chance that two specimens are related). Jacobson et al. 2022 [9] demonstrated that Barratt’s heuristic matrix calculation was the best performing approach when compared to established distance-based approaches. This is likely down to the ability of Barratt’s heuristic approach to account for heterozygosity, integrate the entropy of different markers being analyzed, calculate distance differently depending on whether mitochondrial or nuclear markers were being analyzed, and impute missing data – all of which are advantageous for analyzing sexually-reproducing parasites like *Cyclospora*.

The resulting distance matrix is then passed to a novel clustering algorithm

*Clustering*

The distance matrix is hierarchically clustered and the tree is dissected at a height determined by a stringency parameter and cutoff distance threshold. This approach is described in Jacobson et al. 2023 [10] . To summarize, the distance matrix is cut into partitions so that the smallest partition has a minimum of 2 specimens. The resulting partitions are stored in a list and we randomly select 2 specimens from each partition to build a matrix, where we extract the pairwise distances for each of the selected specimens from the original distance matrix. We then sort the pairwise distances from smallest to largest and select the pairwise distance at the lower 5^th^ percentile. We repeat this process 1000 times to result in a list of pairwise distances at the lower 5^th^ percentile, and we take the average of this list as our cutoff distance. The stringency parameter (in this case 99.5% for 2021) is then applied to our final tree dissection step, where 99.5% of isolates in a given cluster must fall under the cutoff distance threshold.

**References for Supplemental Text**

(1) **Bushnell B.** BBTools: a suite of fast, multithreaded bioinformatics tools designed for analysis of DNA and RNA sequence data. *Joint Genome Institute* 2018.

(2) **Li H, Durbin R.** Fast and accurate short read alignment with Burrows–Wheeler transform. *bioinformatics* 2009; **25**(14): 1754-1760.

(3) **Li H, et al.** The sequence alignment/map format and SAMtools. *bioinformatics* 2009; **25**(16): 2078-2079.

(4) **Langmead B, Salzberg SL.** Fast gapped-read alignment with Bowtie 2. *Nature methods* 2012; **9**(4): 357-359.

(5) **Lindenbaum P.** Jvarkit: java utilities for bioinformatics. *Github* 2015.

(6) **Fu L, et al.** CD-HIT: accelerated for clustering the next-generation sequencing data. *Bioinformatics* 2012; **28**(23): 3150-3152.

(7) **Altschul SF, et al.** Basic local alignment search tool. *Journal of molecular biology* 1990; **215**(3): 403-410.

(8) **Nascimento FS, et al.** Evaluation of an ensemble-based distance statistic for clustering MLST datasets using epidemiologically defined clusters of cyclosporiasis. *Epidemiol Infect* 2020; **148**: e172.

(9) **Jacobson D, et al.** Evaluation of various distance computation methods for construction of haplotype-based phylogenies from large MLST datasets. *Molecular Phylogenetics and Evolution* 2022; **177**: 107608.

(10) **Jacobson D, Barratt J.** Optimizing hierarchical tree dissection parameters using historic epidemiologic data as ‘ground truth’. *Plos one* 2023; **18**(2): e0282154.
